# NMR Methods for Structural Characterization of Protein-Protein Complexes

**DOI:** 10.3389/fmolb.2020.00009

**Published:** 2020-01-28

**Authors:** Jeffrey A. Purslow, Balabhadra Khatiwada, Marvin J. Bayro, Vincenzo Venditti

**Affiliations:** ^1^Department of Chemistry, Iowa State University, Ames, IA, United States; ^2^Department of Chemistry and Molecular Sciences Research Center, University of Puerto Rico, San Juan, Puerto Rico; ^3^Roy J. Carver Department of Biochemistry, Biophysics and Molecular Biology, Iowa State University, Ames, IA, United States

**Keywords:** solvent-PRE, residual dipolar couplings, chemical shift perturbations, solid state NMR, isotopic labeling

## Abstract

Protein-protein interactions and the complexes thus formed are critical elements in a wide variety of cellular events that require an atomic-level description to understand them in detail. Such complexes typically constitute challenging systems to characterize and drive the development of innovative biophysical methods. NMR spectroscopy techniques can be applied to extract atomic resolution information on the binding interfaces, intermolecular affinity, and binding-induced conformational changes in protein-protein complexes formed in solution, in the cell membrane, and in large macromolecular assemblies. Here we discuss experimental techniques for the characterization of protein-protein complexes in both solution NMR and solid-state NMR spectroscopy. The approaches include solvent paramagnetic relaxation enhancement and chemical shift perturbations (CSPs) for the identification of binding interfaces, and the application of intermolecular nuclear Overhauser effect spectroscopy and residual dipolar couplings to obtain structural constraints of protein-protein complexes in solution. Complementary methods in solid-state NMR are described, with emphasis on the versatility provided by heteronuclear dipolar recoupling to extract intermolecular constraints in differentially labeled protein complexes. The methods described are of particular relevance to the analysis of membrane proteins, such as those involved in signal transduction pathways, since they can potentially be characterized by both solution and solid-state NMR techniques, and thus outline key developments in this frontier of structural biology.

## Introduction

The function and survival of cellular organisms are reliant on the ability of cell societies to transfer essential information through communication networks commonly referred to as signaling pathways. The resulting cellular responses of such pathways are mediated by numerous biomolecular interactions that are crucial for regulating various vital biological processes including signal transduction, gene regulation, enzyme catalysis, immune response, signal processing, encoding, and integration (Hunter et al., 2000; Wong and Scott, 2004; Kholodenko, 2006; Anglister et al., 2016). Several important pathologies such as cancer, chronic inflammatory syndrome, and diabetes are commonly dependent upon the malfunction of one or more steps within a signaling pathway (Yarden and Sliwkowski, 2001; Fischer et al., 2003; Gray et al., 2003; Solinas et al., 2007; Wang et al., 2013; Vlahopoulos et al., 2015). Obtaining an atomic-resolution understanding of the dynamic protein-protein interactions underlying regulation of signal transduction pathways is therefore crucial toward the design of effective strategies for therapeutic intervention against human diseases.

Here, we will describe modern NMR methodologies for the characterization of the structure and thermodynamics of protein-protein interactions. This contribution is intended for non-NMR specialists, therefore it is limited to the most common NMR methods for the investigation of protein-protein interactions. In particular, we will introduce various techniques for defining binding interfaces and determining dissociation constants (*K*_*D*_), in addition to other pertinent experiments for NMR structure determination of protein-protein complexes. NMR is uniquely suited to provide atomic-resolution information on the structure, dynamics, and thermodynamics of protein-protein complexes under nearly physiological conditions. The continuous technological advances in solution and solid state NMR are establishing NMR methods as fundamental investigation tools for obtaining insights into the biochemistry of signal transduction pathways.

## Interface and Affinity of Binding Via Solvent Paramagnetic Relaxation Enhancement and Chemical Shift Perturbations Experiments

Targeting specific protein-protein interactions for regulation and inhibition purposes offers a viable way to control and manipulate selective pathways. To capitalize on this approach, it is essential to identify well-defined binding interfaces and binding affinities, which are insightful when developing tactics to modulate protein-protein interactions. NMR is capable of detecting changes in the local electronic environment provoked by binding events, elucidating the regions of a protein involved in a binding interface. Analysis of the NMR data can also provide thermodynamic information on the interaction, such as the binding affinity of a protein-protein complex. Of the available NMR techniques, Chemical Shift Perturbation (CSP) and solvent Paramagnetic Relaxation Enhancement (solvent-PRE) experiments have found widespread use for uncovering details of protein-protein interactions.

### Chemical Shift Perturbation (CSP)

CSP analysis is probably the most informative and widely applicable NMR method utilized for investigating binding interactions (Williamson, 2013; Furukawa et al., 2016). The chemical shift of NMR active nuclei is extremely sensitive to their local electronic environment, which is often perturbed by binding events. The analysis of the change in chemical shift induced by protein-protein binding affords a wealth of information regarding the interaction site and binding affinity.

**Figure 1:**
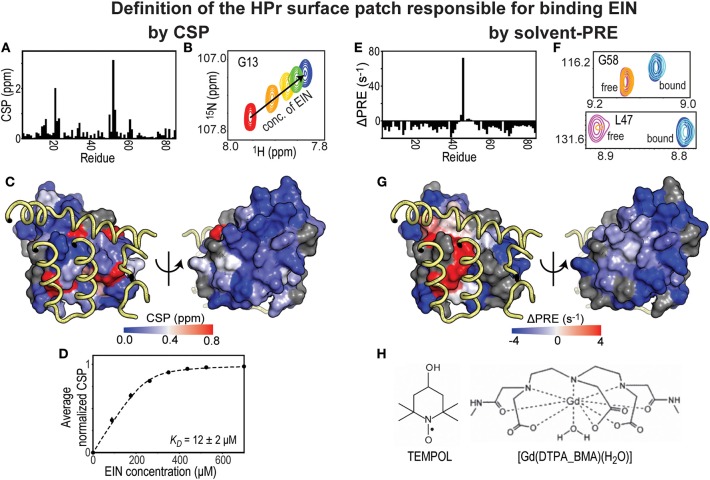
Solvent-PRE and CSP analysis of EIN-HPr complex. **(A)** CSP measured for ^15^N-labeled HPr in the presence of saturating concentrations of unlabeled EIN are plotted vs. residue index. **(B)** Example cross-peak from ^1^H-^15^N HSQC spectrum of ^15^N-labeled HPr measured at increasing concentration of EIN. A peak from the complex interface was selected. **(C)** The CSP data from panel **(A)** are plotted on the surface of HPr according to the color bar. The relevant portions of EIN are shown as yellow tubes. **(D)** CSP vs. concertation of EIN (black circles). The data can be fit (black line) to return the *K*_*D*_ of the EIN-HPr complex. **(E)** ΔPRE vs. residue index. ΔPREs are calculated by subtracting the solvent-PREs (i.e., the increase in ^1^H_N_-*R*_2_ caused by addition of 4 mM Gd(DTPA-BMA) to the NMR sample) measured for ^15^N-labeled HPr complexed to unlabeled EIN from the solvent-PRE data measured for the free protein. While the majority of the HPr residues show a negative ΔPRE (which is the result of the reduced rotational diffusion of complexed HPr compared to the free protein), obstruction of the paramagnetic probe from the binding interface results in positive ΔPREs. **(F)** Example cross-peaks from ^1^H-^15^N HSQC spectra of 0.8 mM ^15^N-labeled HPr in the presence of 0 mM EIN and 0 mM Gd(DTPA-BMA) (pink), 0 mM EIN and 4 mM Gd(DTPA-BMA) (orange), 1 mM EIN and 0 mM Gd(DTPA-BMA) (blue), 1 mM EIN and 4 mM Gd(DTPA-BMA) (cyan). The two cross-peaks have been chosen to illustrate the cases of a residue located far from the complex interface (G58) and of an HPr residue that is in direct contact with EIN (L47). **(G)** ΔPREs are plotted on the surface of HPr according to the color bar. The relevant portions of EIN are shown as yellow tubes. **(H)** Structures of two commonly used paramagnetic probes for surface accessibility studies.

In a typical CSP experiment, a reference 2D-heteronuclear single quantum coherence (HSQC) spectrum of a ^15^N- or ^13^C-labeled protein is acquired in the absence of its binding partner followed by a series of HSQC spectra measured at increasing concentrations of unlabeled ligand (Figures 1A,B). These NMR titration methods are ideally suited, and yield the best results, for weak binding interactions (affinity in the μM-mM range) that exchange between the free and ligated form rapidly on the NMR timescale (i.e., exchange rate ≥ μs^−1^). Indeed, for binding in this fast exchange regime, the observed chemical shifts are a population weighted average of the chemical shifts of the free and complexed protein (Williamson, 2013; Furukawa et al., 2016). Therefore, a plot of the chemical shift change as a function of the concentration of binding partner results in a binding isotherm that can be fit to obtain the dissociation constant (*K*_*D*_) for the protein-protein complex (Figures 1B,D). Mapping CSP at a saturating concentration of binding partner on the structure of the protein observed by NMR provides information on the residues that reside at the interface of the complex (Figure 1C). However, protein-protein interfaces highlighted by CSP are usually more ambiguous than the ones detected by solvent-PRE experiments (Figures 1C,G). Indeed, intermolecular contacts involving atoms on long side-chains may not produce substantial changes in the electronic environment of the corresponding backbone amide, and go undetected by CSP (which are conventionally measured using ^1^H-^15^N HSQC). In addition, unlike solvent-PRE, CSP data are extremely sensitive to allosteric conformational changes that can occur upon binding (Boulton and Melacini, 2016). In the absence of additional structural information, distinguishing changes in chemical shift due to direct protein-protein contacts from the ones induced by an allosteric conformational change can be a challenging task.

NMR titration experiments are employed also in the investigation of tight protein-protein interactions that fall in the sub-μM to nm affinity range (Williamson, 2013; Furukawa et al., 2016). For such strong binding events, which often undergo intermediate to slow exchange on the NMR timescale, the effect of the titrant on the appearance of the NMR signals is different from the fast exchange case. Indeed, in the slow exchange regime, separate peaks are observed for the free and complexed forms, while the intensity of the NMR peaks will be attenuated in the case of intermediate exchange (note that the maximum attenuation is expected for the middle point of the protein-protein titration experiment). Therefore, determining the *K*_*D*_ for such systems by NMR can be difficult as the dependency of the NMR signal intensity and chemical shift on the populations of the free and bound states is not trivial. NMR methods combining *R*_2_ relaxation dispersion and ZZ-exchange measurements have been developed to obtain kinetic, thermodynamic, and structural information on binding events occurring on the intermediate and slow exchange regimes (Furukawa et al., 2016).

### Solvent-Paramagnetic Relaxation Enhancement (PRE)

Solvent-PRE effects arise from the magnetic dipolar coupling between an NMR active nucleus on the protein under investigation and one (or more) unpaired electron(s) located on a paramagnetic molecule used as a solvent accessibility probe. The nucleus-electron coupling effectively enhances the longitudinal and transverse nuclear spin relaxation rates (*R*_1_ and *R*_2_, respectively) by an amount that is proportional to the local concentration of the paramagnetic molecule (Varrazzo et al., 2005; Bernini et al., 2009). Solvent-PREs are routinely measured by taking the difference between the ^1^H-*R*_2_ rate measured in the presence of the paramagnetic probe and the ^1^H-*R*_2_ rate measured in a diamagnetic reference sample (Figures 1E,F) (Anthis and Clore, 2015). Consequently, in the case of a folded globular protein, solvent-PREs are expected to decrease with increasing distance from the molecular surface (Bernini et al., 2009). For identifying a protein-protein binding interface, solvent PREs are measured for the free and complexed forms, where the primary protein of interest is NMR visible (i.e., ^15^N and/or ^13^C labeled) and the other is NMR invisible (i.e., at natural isotopic abundance). Upon protein-protein complex formation, the previously exposed binding surface will become internally buried, effectively decreasing the solvent-PRE measured for the nuclei at the binding interface (see Figures 1E–G) (Arumugam et al., 1998; Bernini et al., 2006a; Garimella et al., 2006). In theory, the same protocol may be repeated upon reversing the labeling scheme to pinpoint residues from the seconds protein that reside at the interface of the complex.

From the practical point of view, it is important that the paramagnetic probe does not establish electrostatic and/or hydrophobic interactions with the investigated proteins. This condition eliminates the possibility of a biased distribution of collisions between the small molecule probe and the macromolecular surface, therefore permitting a direct interpretation of the solvent-PRE data in terms of solvent accessibility. Among the commercially available small paramagnetic molecules, TEMPOL and Gd(DTPA-BMA) have been reported to show minimal interactions with proteins and nucleic acids, and have been employed to characterize the surface accessibility of several macromolecules and macromolecular complexes (Figure 1H) (Pintacuda and Otting, 2002; Venditti et al., 2007, 2008; Staple et al., 2008; Hartlmuller et al., 2019). In some reports, the solvent-PRE produced by multiple paramagnetic probes are analyzed simultaneously to exclude the existence of preferential probe-macromolecule interactions and obtain an accurate picture of the macromolecular surface (Bernini et al., 2006b). Albeit solvent-PRE experiments provide a wealth of structural information on macromolecular complexes, a major drawback is their inability of extracting thermodynamic parameters on protein-protein interactions. CSP experiments can be employed to fill this gap.

### Structural Models From Solvent-PRE and CSP Data

Albeit CSP and solvent PRE analysis are powerful tools for distinguishing binding interfaces, structure-activity relationships, and *K*_*D*_ values (Nerli et al., 2018; Nitsche and Otting, 2018), additional structural information on the protein-protein complex can be derived by combining CSP and/or solvent-PRE data with molecular docking simulations. There is now available software which provides a straightforward approach for defining complex structures from integrated solvent-PRE and/or CSP datasets using docking simulations (Dominguez et al., 2003; Madl et al., 2011).

## NMR Techniques for Determination of Atomic Resolution Structures of Protein-Protein Complexes

The solvent-PRE and CSP experiments discussed above are simple and inexpensive techniques that are ideally suited for low-resolution studies of protein-protein interactions. Obtaining a higher resolution look into macromolecular complexes requires detailed information on the specific interatomic contacts across the complex interface and on the relative orientation between the binding partners. Other NMR observables, such as nuclear Overhauser effect (NOE) and Residual Dipolar Coupling (RDC), are commonly employed for more advanced studies on protein-protein interactions.

### Intermolecular Nuclear Overhauser Effect (NOE)

The determination of 3D, atomic-resolution structures of macromolecules by NMR traditionally relies on the measurement of interproton distances from NOE experiments (Clore and Gronenborn, 1998). NOEs can be detected between protons that are in spatial proximity (distance < 6 Å) within the macromolecule, and therefore provide short interproton distance restraints for structure calculation protocols. A major drawback of using NOE experiments in the investigation of protein-protein complexes is their *r*^−6^ dependency on the internuclear distance, which makes intermolecular NOEs much weaker and harder to observe than intramolecular NOEs. To overcome such limitation, many NMR methods have been introduced to purge intramolecular NOEs and selectively observe intermolecular dipolar couplings (Anglister et al., 2016). Among these methods, isotope-edited/isotope-filtered experiments are routinely employed in the analysis of protein-protein interfaces.

**Figure 2:**
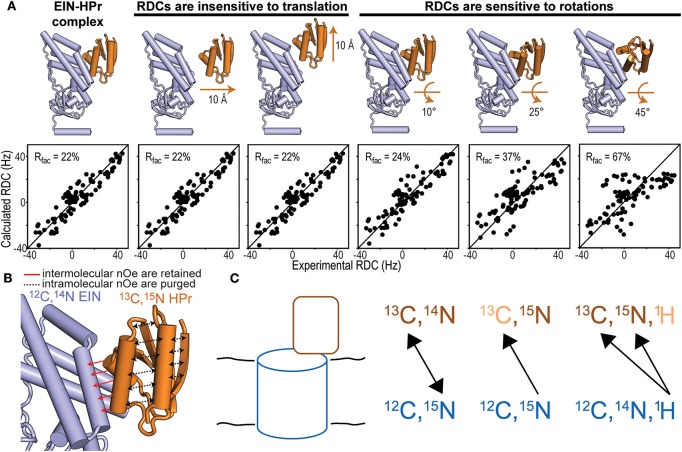
NMR methods for structure determination of macromolecular complexes. **(A)** Fitting of the experimental N-H_N_ RDCs measured for the phosphorylated EIN-HPr complex (Suh et al., 2008) to (i) the experimental NMR structure (PDB code: 3EZA; left panel), (ii) two structural models generated by 10 Å translation of HPr along two perpendicular directions (seconds and third panels from left), and (iii) three structural models generated by rotations of 10°, 25°, and 45° of HPr about an axis perpendicular to the complex interface (last three panels from left). The quality of the fit is judged in terms of R-factor. A high R-factor indicates poor agreement between the experimental RDC data and the structural model (Clore and Garrett, 1999). **(B)** In an isotope-edited/isotope-filtered experiment intramolecular NOEs (black dashed lines) are purged, while intermolecular NOEs (red solid lines) are retained. In panels **(A,B)** HPr is orange and EIN is blue. **(C)** Schematic representation of a solid protein complex and heteronuclear polarization transfer schemes. In the first scheme, mixtures of isotopic labeling allow intermolecular heteronuclear correlations. In the seconds scheme, intramolecular ^15^N-^13^C dephasing precedes intermolecular heteronuclear ^15^N-^13^C correlations. In the third scheme, intramolecular ^1^H^11^H-^13^C^33^C/^15^N^55^N dephasing facilitates intermolecular ^1^H-^13^C/^15^N cross-polarization that identifies isotopically labeled sites on a protein interacting with an unlabeled binding partner.

Isotope-edited/isotope-filtered pulse sequences utilize an initial INEPT (or HMQC) pulse train to select the magnetization originating from protons covalently bonded to ^13^C- or ^15^N-labeled nuclei. This step is referred to as isotope-editing and has the effect of retaining only the magnetization originating from an isotopically enriched protein. During the subsequent mixing period, the longitudinal magnetization prepared by the isotope-editing step is transferred to nearby protons by NOE. The final isotope-filtering step eliminates the magnetization from protons attached to ^13^C- or ^15^N-labeled heteronuclei. Therefore, if proteins A and B in the binary complex are ^13^C/^15^N- and ^12^C/^14^N-labeled, respectively, the NMR signal detected by an isotope-edited/isotope-filtered experiment reports on NOE transfers between the ^13^C and ^15^N bonded protons on protein A and the ^12^C and ^14^N bonded protons of protein B (Figure 2B). Indeed, the editing and filtering steps of the pulse sequence ensure that intramolecular NOE effects, which would obscure the weaker intermolecular couplings, are effectively purged out of the detected NMR signal (Anglister et al., 2016).

Isotope-edited/isotope-filtered NMR experiment have been used to investigate intermolecular interactions in relatively large (up to ~70 kDa) complexes and macromolecular assemblies (Garrett et al., 1999; Williams et al., 2004; Xu et al., 2006). Applications to larger systems are in principle possible, especially if combined with isotope-labeling schemes that reduce the proton transverse relaxation rate. Selective ^13^CH_3_ labeling of Ile, Leu, and Val side-chains in an otherwise perdeuterated background can potentially push the molecular weight limit in the >100 kDa range (Tugarinov et al., 2003). However, as this labeling scheme dramatically decreases the number of expected intermolecular dipolar couplings (note that perdeuteration effectively reduces the number of protons at the complex interface), additional experimental restraints need to be employed to accurately determine the 3D structure of the macromolecular complex.

### Residual Dipolar Coupling (RDC)

RDCs are NMR observables that are commonly employed to acquire long-range structural restraints for structure calculation protocols (Prestegard et al., 2000; Venditti et al., 2016). In the vast majority of applications, RDCs are measured for covalently attached NH or CH groups and provide information on the angle formed by the N-H or C-H bond vectors with the external magnetic field (Tjandra and Bax, 1997). RDCs are especially powerful for determining the relative orientation of two proteins within a complex as, if the proteins can be treated as rigid bodies, only a small number of RDCs are required to fulfill the geometric problem (Clore, 2000). It is important to stress out that, while RDCs are highly sensitive to rotations, they are completely insensitive to translational motions (Figure 2A). Therefore, 3D structures of macromolecular complexes cannot be solved using exclusively RDC data. Accurate structures of large protein–protein complexes can be obtained on the basis of few intermolecular NOEs to provide translational information, supplemented by RDCs for orientation. This approach has been employed to resolve the atomic resolution structures of all protein-protein complexes mediating signaling in the bacterial phosphotranspherase system (Clore and Venditti, 2013).

A complication in the application of RDCs to the investigation of protein-protein complexes is that the experiment requires the molecular complex to be partially aligned with the magnetic field during the acquisition of the NMR data. This condition involves preparation of the NMR sample in a dilute liquid crystalline medium such as bicelles (Tjandra and Bax, 1997) or phage (Hansen et al., 1998). While it is very important that the alignment medium employed for the measurement does not perturb the structure of the complex under investigation, several protocols for preparation, and optimization of alignment media have been reported in the literature to facilitate this endeavor (Venditti et al., 2016).

## Protein-Protein Interactions in the Solid State

Very large biomolecular complexes, such as protein fibrils, microtubules, virus particles, or membrane proteins in a native-like lipid bilayer, constitute systems that restrict molecular tumbling and thus behave as solids. Solid-state NMR spectroscopy reintroduces the resolution otherwise lost due to the orientation-dependent nature of nuclear magnetic interactions. Magic-angle spinning (MAS) NMR permits the complete assignment of ^13^C and ^15^N resonances for small and medium-sized proteins. Using perdeuteration or ultrafast MAS, ^1^H assignments can also be obtained. Therefore, MAS NMR facilitates the atomic-level analysis of protein-protein interactions in biological solids (Marulanda et al., 2004; Miao and Cross, 2013; Arachchige et al., 2018; van der Wel, 2018). Systems where only one of the two interacting proteins is natively found in the solid-state, as is the case with membrane proteins that interact with soluble proteins as part of a signal transduction pathway, are also amenable to solid-state NMR analysis. Figure 2C depicts such case, in which solid-state NMR spectra with and without the peripherally attached protein can be recorded to identify binding interfaces in the membrane protein via CSPs. On the other hand, characterizing the binding interface of the soluble protein in this complex requires resonance assignment for this protein in the bound state, precluding CSP analysis. Indeed, solid-state samples present different challenges but also opportunities for detailed understanding of protein complexes in native-like environments.

Solid-state NMR analysis of protein-protein interactions is guided in part by some of the concepts described above for solution NMR, including solvent PRE and CSPs, which can often be applied in similar manners in both solution and solid-state NMR experiments (Wang et al., 2012; Park et al., 2015; Dannatt et al., 2016; Rogawski and McDermott, 2017; Theint et al., 2018). However, the efficiency and flexibility of heteronuclear dipolar recoupling in MAS NMR, where interactions between different types of nuclei can be selectively reintroduced, facilitates the implementation of intermolecular polarization transfer approaches analogous to the intermolecular NOE in solution NMR, but leveraging the selectivity of ^15^N-^13^C or ^1^H-^15^N/^1^H-^13^C dipolar couplings. Intermolecular polarization transfer simultaneously identifies binding interfaces and elucidates structural constraints in an unambiguous manner. The choice of intermolecular polarization transfer scheme depends on the ability to isotopically label the proteins involved.

### Intermolecular Heteronuclear Recoupling

In cases where both interacting proteins can be independently produced and isotopically labeled, one of the more versatile approaches is to analyze a sample when one protein (^12^C, ^15^N) is labeled uniformly with ^15^N and the other protein (^13^C, ^14^N) is labeled uniformly with ^13^C (Yan et al., 2013; Demers et al., 2018). Dipolar recoupling experiments can then be applied to record a series of 2D ^15^N-^13^C correlation spectra with increasing mixing times that provide distance-dependent cross-peak intensities from which internuclear distances can be estimated. Alternatively, a single long-range mixing spectrum can be used to obtain qualitative information based on the relative signal intensities between the different cross-peaks. Thus, each ^15^N-^13^C pair corresponds to an intermolecular contact whose identification depends on having obtained resonance assignments for each protein. Since ^15^N resonance lines typically display modest resolution, identifying multiple contacts in the same region often supports unambiguous assignment of the intermolecular cross-peak. The experiment can be repeated for a sample consisting of the proteins labeled in reversed fashion to record a complementary set of intermolecular ^15^N-^13^C correlations. Heteronuclear techniques such as TEDOR and PAINCP have been implemented to obtain long-range correlations of such mixed-labeled samples (Yang et al., 2008). The spectral simplification of intermolecular spectra facilitates the application of dynamic nuclear polarization to enhance experimental sensitivity (Bayro et al., 2011).

### Dephasing Methods

Filtering signals via heteronuclear dephasing allows the use of samples where one protein is uniformly ^13^C and ^15^N labeled and the other protein is only ^15^N labeled. One-bond ^15^N-^13^C REDOR dephasing eliminates intramolecular correlations, which is then followed by long-range heteronuclear polarization transfer to generate intermolecular ^15^N-^13^C correlations. For the transfer step, PAINCP is an effective choice that exploits higher-order heteronuclear coherences (Yang et al., 2008). This diphase-transfer approach has the advantage of permitting sequential assignment and assignment verification for the uniformly ^13^C, ^15^N labeled protein with the same sample.

### Intermolecular Cross-Polarization

Polenova and coworkers recently demonstrated an approach in which a uniformly ^13^C, ^15^N labeled protein can be analyzed in complex with a natural abundance protein (Guo et al., 2017). First, ^1^H-^13^C (or ^1^H-^15^N) interactions are dephased in the labeled protein, and then ^1^H-^13^C (or ^1^H-^15^N) cross-polarization is used to transfer polarization between ^1^H nuclei in the unlabeled protein and ^13^C (or ^15^N) nuclei in the labeled protein. This approach allows the identification of the binding surface of a protein in complex with another protein or protein assembly that cannot be isotopically labeled efficiently, thus enhancing the range of applications possible with solid-state NMR methods for protein-protein interactions.

## Conclusions

Signaling pathways are sophisticated communication networks that play a fundamental role in controlling virtually all cellular responses through various protein-protein interactions. By virtue of their significant biological relevance, such interactions have generated substantial interest for their potential use in the treatment of correlated human pathologies. With this aim, it is imperative to gain an explicit characterization of specific protein-protein interactions. Albeit there are several techniques applicable for investigating macromolecular systems, NMR has a unique ability of providing thermodynamic and structural information on macromolecular complexes with atomic-resolution. Indeed, the combination of solvent-PRE and CSP experiments permits the extraction of well-defined binding interfaces, affinities, and binding modes. NOE and RDC data are exceptionally valuable for determining crucial interatomic contacts and structural orientations for protein-protein interactions. Further, additional NMR experiments, such as DEST, relaxation dispersion, PRE, and PCS (not described in this contribution), in combination with sophisticated isotopic labeling techniques have been employed to characterize a number of high molecular weight protein-protein complexes (Libich et al., 2013, 2015; Anthis and Clore, 2015; Danilenko et al., 2019). The exhaustive knowledge of the molecular mechanisms driving formation and stabilization of protein-protein complexes provided by NMR analysis (often supplemented by other investigation techniques) is a fundamental step toward establishing effective strategies for manipulation and control of specific intermolecular interactions within signaling pathways.

## Data Availability Statement

The datasets generated for this study are available on request to the corresponding author.

## Author Contributions

JP, BK, MB, and VV wrote the manuscript. MB and VV obtained research funding.

### Conflict of Interest

The authors declare that the research was conducted in the absence of any commercial or financial relationships that could be construed as a potential conflict of interest.
